# Supplementary material for: Vampyrum spectrum (Phyllostomidae) movement and prey revealed by radio-telemetry and DNA metabarcoding
Source: PLoS One. 2022 Apr 5;17(4):e0265968. doi: 10.1371/journal.pone.0265968 (PMC8982828; doi:10.1371/journal.pone.0265968)
Supplement: Attachment — Submitted filename: Response to Reviewers.docx [file pone.0265968.s002.docx]

Response to Reviewers

Review Comments to the Author

Please use the space provided to explain your answers to the questions above. You may also include additional comments for the author, including concerns about dual publication, research ethics, or publication ethics. (Please upload your review as an attachment if it exceeds 20,000 characters)

Reviewer #1:

**This research is a great contribution to the knowledge base of a rare and carnivorous bat species, Vampyrum spectrum. The article is well written, and I would like to see a bit more detail in the Methods so that others could replicate the steps taken to analyze fecal samples and to find the roosting tree. For publication in PLOS ONE, this paper could also benefit from some discussion of other carnivorous bat studies, particularly those using eDNA. Adding some additional supporting citations could help reach a broader audience. I also recommend a slight change to the title that better represents the underlying data.**

We have made changes throughout the manuscript to address all suggestions. See responses to particular questions bellow.

**Line 4: Space use may infer home range or foraging range analysis; in this study, there were two relocations (capture site, dropped transmitter) from one individual. Whereas these data points shed some light on two specific foraging locations, they are a very small proportion of the data needed to estimate space use.
Suggest changing ‘space use’ to ‘foraging distance’ or ‘foraging activity’ in the title.
• Also capitalize each word in title**

Title was modified and words capitalized as suggested.

**Line 25: add ‘is’ before ‘listed as’**

Changed as suggested

Line 34: The prior line states that primers were used to detect vertebrates and arthropods, but then there is no information about arthropods detected. Were there any obvious large arthropods detected such as katydids or beetles? Even if these weren’t quantified (as stated in the Results section), it would be worth noting here for comparison with other carnivorous bat studies.

We have expanded on our reasoning for why we decided to not comment on arthropods/invertebrates. Due to a combination of incomplete reference libraries of invertebrates from the Central American region and Neotropics, confidence in ID to species level was low. As reference libraries become more complete, we believe all raw sequences we are making available in NCBI’s depositories will allow a future re-analysis of the data and provide much more informative results.

Please see also our responses for comments on line 136 and 233.

**Line 39: Change ‘the species’ to ‘Vampyrum spectrum’ for a stronger closing**

Changed as suggested

**Line 47: Change ‘associated with’ to ‘inhabit’**

Changed as suggested

**Line 49: Change ‘through’ to ‘throughout’**

Changed as suggested

**Line 60: Reword this sentence for flow: ‘Feathers found at a communal roost entrance suggest the predation and transport of at least 18 bird species ranging from the 150 g White-tipped Dove (Leptotila verreauxy) to the 20-g Banded Wren (Thryothorus pleurosticus) [2].• Also, capitalize all bird species common names throughout (line 259)**

Sentence reworded as suggested and all bird species’ common names are capitalized now.
 **Line 66: ‘often implies that collection of live specimens is necessary’**

Changed as suggested

**Line 70: Add a paragraph about what is known about the diet of neotropical carnivorous bats from metabarcoding studies (e.g., Jones, P. L., Divoll, T. J., Dixon, M. M., Aparicio, D., Cohen, G., Mueller, U. G., ... & Page, R. A. (2020). Sensory ecology of the frog-eating bat, Trachops cirrhosus, from DNA metabarcoding and behavior. Behavioral Ecology, 31(6), 1420-1428.)**

We added as suggested

**Line 71: add ‘to’ before describe; ‘activity patterns’ is a bit vague, consider changing to ‘foraging distance’ or ‘foraging areas’**

Changed as suggested

**Line 74: delete ‘for foraging’, add ‘while foraging’ to end of sentence**

Changed

**Line 81: Approximately how many km wide and long is the Rivas Isthmus? This info will help readers visualize the broader landscape**

Information added (18 km)

**Line 83: Add info about elevation changes or landscape features. I realize it is lowland, but any karst features may make it more challenging to conduct radio telemetry. A bit more detail here could give readers an appreciation for the challenges of tracking bats in that area.**

Information added

**Line 98: With PLOS ONE’s broad readership, it would help to add ‘skin’ or ‘medical’ before the word glue, for those unfamiliar with the common practice of using this type of glue on animals.**

Word “medical” was added

**Line 99: Is there a citation or some more detail about the ‘close approach method’? More detail will allow others to replicate the method when searching for similar roost types.**

**Line 100: Was a 3- or 5-element yagi antenna used? This will help others choose the right equipment**

Information added (2 elements)

**Line 107: Describe the start and end time of the exit/entry surveys so readers can guage whether the bats are going out/in several times per night or for one extended foray.**

Information added

**Line 110: Consider an alternate term to ‘≥24 hr’. Is it possible that these samples were defecated less than 24 hr before collection? For example, most of the sample might be days or weeks old, but some might have been deposited 15 min before the samples were collected. Maybe ‘roost sample’ and ‘fresh sample’?**

We renamed and defined samples collected upon discovery of the roost and after as “Sample A” and “Sample B”

**Line 127: Were samples indexed on both ends and were these prepared for paired-end sequencing? How many samples were run simultaneously and were samples from all primer sets combined in the same run?**

We added some details on the DNA analysis section in Methods; however, specific information about methods were already provided in references cited (i.e., reference 37).

**Line 131: Add some detail about the parameters chosen during bioinformatics. What quality threshold was used for trimming? What copy number threshold was used to determine if OTUs were potential sequencing artifacts? Was there a step to check for chimeras or crosstalk between samples? (Schnell, I. B., Bohmann, K., & Gilbert, M. T. P. (2015). Tag jumps illuminated–reducing sequence-to-sample misidentifications in metabarcoding studies. Molecular Ecology Resources, 15, 1289–1303.** [**https://doi**](https://doi)**. org/10.1111/1755-0998.12402)**

Additional details about bioinformatic parameters and quality control were added on the DNA analysis section in Methods.

**Line 136: Were taxonomic assignments always clear cut to another sister species or backed off to genus in ambiguous cases (i.e., more than one sister species possible in the same genus)?**

All items reported at the species level were clear cut matches. On a few occasions the closest match was to a sister species that did not occur in our study area. Taxonomical splits and lack of genetic references from Nicaraguan populations were the main reason of mismatches. Taxonomy was updated in Table 2 to reflect the match of our samples with the GenBank reference sequences.

**Line 152: What happened between Mar. 4 and Mar. 14? Was the bat elsewhere or not searched for during that timeframe?**

We monitored the bat from Mar 5 but we were not able to climb the tree and pin point the roost entrance until March 14. A clarification was added.

**Line 165: ‘due to decomposition’**

Changed as suggested

**Line 201: add ‘which range from’ after Gallus gallus to avoid confusion. At first I thought that the results indicated chickens of various sizes in the diet of the bats.**

Changed as suggested

**Table 2: Use en dashes between the values in the Mass column**

Changed as suggested

**Line 233: Were there any unusually large arthropods detected, such as beetles or katydids? The primer sets should pick them up and it would be worth noting, even if not quantified, for comparison with other studies that have documented V. spectrum eating insects (Bonato et al. 2004).**

The incompleteness of reference sequence on GenBank for arthropod species in the neotropics was another reason for not expanding on the arthropod diet of *V. spectrum*. We manually BLASTED all sequences from all markers and no there were no matches for large arthropods at least with the current libraries. We added this comments in this section of the manuscript.

**Line 251: Perhaps note that ANML was the only set to detect the screech owl? Hence, it may be worth using multiple primer sets, when possible, to recover more prey items than one primer set alone.**

Comment was added as suggested

**Line 275: change ‘were’ to ‘are’ when referencing published findings**

Changed as suggested

**Line 282: The interesting things about V. spectrum foraging opportunistically on small vertebrates is that they don’t appear to eat anurans or lizards like other carnivorous false-vampires (False Vampires and Other Carnivores - Bat Conservation International). Do you expect that the primer sets would have picked up reptiles if they had eaten them? Some comparison/discussion of the diet of other false vampires (at least neotropical, and possibly African and Australian) would be useful here and appeal to a broader audience.**

Yes, the 12S and 18S marker used should have been capable of picking up reptiles or anurans. We added a paragraph expanding on this idea.

**Line 296: Sheppard et al. 2005 describe this secondary predation phenomenon: Sheppard, S. K., Bell, J., Sunderland, K. D., Fenlon, J., Skervin, D., & Symondson, W. O. C. (2005). Detection of secondary predation by PCR analyses of the gut contents of invertebrate generalist predators. Molecular ecology, 14(14), 4461-4468**.

We added this reference and expanded on why we decide not to report arthropods on this study.

**Line 298: ‘longer period ,and, thus’**

Added a comma

**Line 300: delete duplicate ‘preserve’**

Deleted

**Line 314: Add citation for this statement. See Arrizabalaga-Escudero, A., Garin, I., García-Mudarra, J. L., Alberdi, A., Aihartza, J., & Goiti, U. (2015). Trophic requirements beyond foraging habitats: The importance of prey source habitats in bat conservation. Biological Conservation, 191, 512-519.**

Citation added

**Line 315: Clarify ‘forest management’ here. Some may assume that forest management = timber harvest, which sounds at odds with mature forests that V. spectrum may require. Are there any management practices that might support large roost trees in mature forest?**

Changed to “habitat management and conservation” for clarity

**References: check italics on species names and en dashes in page number ranges. Also add missing DOIs**

We double checked the completeness of the reference and added DOIs. We used a reference manager (Mendeley) and “Vancouver” style for formatting the reference as indicated by the author guidelines. Some literature that we cite lack DOIs.

Reviewer #2:

I assumed by the title that the manuscript would give details about space use by V. spectrum. However, the manuscript describes the time one individual leaves and arrives to the roost, and we have only three locations: capture, roost and one location where the transmitter was dropped. The radiotelemetry was used to find the day roost, in which the feces were collected and the diet was accessed by DNA metabarcoding techniques.
I liked the relation between the prey habitats and the carnivore habitat, and it can generate important insights about the carnivore niche, especially when it is about a difficult to find and monitor species like V. spectrum.

The manuscript is well written and the research about the natural history of the species is well conducted. I tried to help make the text clearer. Please see my specific comments below.

**Line 52 – localities where V. spectrum was captured?**

Clarification added, Changed to “capture sites”

**Line 56 e 57 – Can you explain more about the relation between foraging activity and resource availability for V. spectrum? As a generalist carnivore, one can assume that this species in not highly impacted by seasonality, for example.**

Although this is an interesting point, we are unclear what the reviewer is suggesting and don’t feel that there is adequate information available on the species to assume it is not impacted by seasonality.

**Line 149 and 150 – I do not understand why it was necessary to use two transmitters in a single individual.**

We opted for two transmitters since previously other larger bats have been able to detach their glued transmitters within a few days. The collar was an experiment and we wanted to increase the opportunity to obtain data for as many days as possible. We added a clarification for this decision in the methods section.

**Line 157 e 158 – The text is “and six Fresh fecal samples.”. But if it was the first time of accessing this roost, the researchers would not have set the plastic to collect fresh fecal sample yet, right?
I found that information on Line 212. Please, add that information on line 157 as well.**

Clarification on this procedure was added

**Line 175 – What do you mean by “opportunistically”?**

We meant that it was conducted within the possibilities of our personnel availability and were not conducted on consecutive nights necessarily. We added a clarification on this sentence.

**Line 186 – here is “lactating female”, but in the line 142 is post-lactating.
Which is correct?**

We corrected it to Post-lactating.

**Line 208 – What defines one fecal sample? If the feces are on the floor and you described above “The guano pile itself was 10 cm deep”.**

In this case we referred as a “fecal sample” a tube with a subsample of ~5ml of fecal matter from the pile. We added this definition to the table legend and methods.

**Table 2: Please, add to the description of the table the meaning of “ANML”, “SF”, “F”, “12S” and “18S”**

This was added to the table title

**Line 256 – 259. I do not understand the sentence.**

We rephrase and restructure the paragraph for clarity.

**Line 270 – 278. It seems to me that the sentences describing bat prey niches are better placed on line 266, after the sentence “Bat species represented most of the mammal species detected in our samples and represented a range of masses, foraging guilds, and habitat associations.”**

Sentences were moved as suggested

**Line 300 – delete first “preserve”.**

Deleted
 **Line 303 – 304. If the female was lactating, the puppy should be with her, right? Why didn`t you see it?**

We believe that one of the individuals is the “puppy” but at this stage is as big as the parents and might already started feeding on captured prey rather than lactating. The female was post-lactating and female bats don’t carry pups every time they fly. We stated this in the results section.
**Line 316 – Please explain more about “Similar patterns for association of roost and foraging habitats”. What association?**

We added a clarification for this

**Line 317 – What species?**

Examples added **Please explain how the activity monitoring was conducted. Does the table 1 describe the first time the individual left the roost during the night? Does the individual leave the roost again after? Do observation nights consisted of being close to the tree recording the time the individual leave and arrive?
The individual was captured on 3 March but the roost was found on 14 March. Would you comment on the difficulty of finding the individual? Was it recorded overnight even without locating the roost?**

We added clarification for all these points in methods and results. The bat monitored left only once per night. We expanded the detail on our methods for activity monitoring in the method section under the ‘live capture and telemetry” sub-heading. We added information on the challenges of tracking the bat in that environment on our results section.
