# Supplementary material for: Vampyrum spectrum (Phyllostomidae) movement and prey revealed by radio-telemetry and DNA metabarcoding
Source: PLoS One. 2022 Apr 5;17(4):e0265968. doi: 10.1371/journal.pone.0265968 (PMC8982828; doi:10.1371/journal.pone.0265968)
Supplement: Attachment — Submitted filename: Response to reviewers.docx [file pone.0265968.s003.docx]

**Responses to Reviewers (second round or revisions)**

Please use the space provided to explain your answers to the questions above. You may also include additional comments for the author, including concerns about dual publication, research ethics, or publication ethics. (Please upload your review as an attachment if it exceeds 20,000 characters)

Reviewer #1: Comments on the tracked changes version:

**1. Line 75 - change "Neotropics DNA techniques" to "Neotropics, DNA metabarcoding"**

Changed as suggested

**2. Line 107 - change "the roost" to "roost sites". In the methods, we don't know yet that there is only one roost**

Changed as suggested

**3. DNA Collection section - The sample numbers were lost in the updates. Specify how many of Sample A and how many of Sample B**

Changed as suggested, subsample numbers were re included in the text.

**4. Line 147 - How many total samples were in the sequencing run, after PCR (including controls) but before equimolar pooling? This will be important for readers to understand how 16 raw fecal samples equals many more after splitting for each primer set and PCR (e.g., were there duplicate samples, were there PCR failures, was there equal representation from all the primer sets). Some studies may run 96 samples for the convenience of using a 96-well plate during PCR and some multiplex many more into one sequencing run; the results may be different for each sampling strategy.**

We added a clarification on this. We used 4 sets of replicates of the original 16 samples to be use with each primer set (4 primers) for a total of 64 replicated samples. Each primer set required different conditions in PCR runs, so we only ran PCRs one replicate-primer at a time on their own plate with their own NTCs and PTCs. If we count the samples and controls, there were a total of 72 data points in 4 plates. We did not experience failures in the PCR runs.

**5. Line 179 - commas before and after "therefore"**

Changed as suggested

**6. Line 274 - this idea needs a bit more clarification. Using the potential to detect prey that arthropods had eaten is not not a strong justification as there may have been secondary detections from birds or bats eating arthropods or secondary bird or mammal detections in vampire bats. I think for this paper it is fine to not report arthropod detections in detail, the justification just needs a bit of clarification. Can you present a proportion of ASVs? If arthropods were a small proportion of ASVs, then that may be justification for not reporting those results. The BOLD database and GenBank have a lot of arthropod sequences for the COI barcode, and though the exact Neotropical species may not be represented, results would come back in the same genera or families. It would be useful to know if there were any arthropods that were detected in most samples or if it was a selection of low frequency results from various arthropod orders.**

We think this is an interesting question but beyond the scope of this paper. We found 13% of ASVs represented arthropods (22 Orders) in our sample. They represented leafhoppers, flies, cockroaches, micro-caddisflies, grass flies, copepods, and moths. However, we did not feel confident concluding they were potential arthropod diet items from *V. spectrum*. We did address the fact that prey diet could be picked up from the host’s fecal material and had already addressed this in the document. We believe that the data on small vertebrates presented in the manuscript summarizes the most likely diet items of our study carnivore species.

 We believe that a comprehensive analysis of arthropods should involve sampling and barcoding of these on our study site. In our manuscript we briefly mentioned arthropod orders found in the samples and published all raw ASV data from all primer sets for future research in this topic (line 261-271; 318-329).

Reviewer #2: This research is a great contribution to the knowledge base of a rare and carnivorous bat species, Vampyrum spectrum. All the comments have been addressed and the manuscript is clear and well-written.
